# Chlamydia trachomatis Infection Could Be a Risk Factor for Infertility in Women: A Prospective Descriptive Study

**DOI:** 10.7759/cureus.30697

**Published:** 2022-10-26

**Authors:** Elena Cortes, Berta Montero, Oriol Yuguero

**Affiliations:** 1 Medicine and Surgery, Universitat de Lleida, Lleida, ESP; 2 Gynecology, Hospital Universitario Arnau de Vilanova, Lleida, ESP

**Keywords:** youth health, sexually transmitted infection (sti), screening, chlamydia trachomatis, women fertility

## Abstract

Objective: To determine whether previous *Chlamydia trachomatis *(CT) infection among sexually active women is significantly associated with a diagnosis of infertility.

Methods: A prospective descriptive study was conducted in Lleida Health Region (Spain). Women who attended medical consultations for infertility at a public university hospital in 2021 were included in the study. Data were collected during January and February 2022 using the hospital's electronic records and clinical interviews.

Results: The study revealed that having immunoglobulin (Ig)G antibodies for CT was associated with an increased rate of infertility compared to patients with negative titers(p-value < 0.05). Age was also associated with infertility. There was no statistically significant difference among the other characteristics studied, such as previous sexually transmitted infections (STI), previous miscarriages, preliminary cervical lesions, and levels of follicle-stimulating hormone (FSH), estradiol, thyroid-stimulating hormone (TSH), prolactin, and anti-mullerian hormone (p-value > 0.05).

Conclusion: The study showed a high prevalence of infertility among women who had IgG CT antibodies. Although more studies should be conducted, promoting strategies among young women to control this infection may help reduce infertility.

## Introduction

The *Chlamydia trachomatis *(CT) bacterium is currently the main cause of sexually transmitted infection (STI) and is the most prevalent infection among young people (both men and women) aged 14 to 25 years. In addition, the majority of these infections occur without any symptoms [1]. This can lead the pathogen to persist and cause long-term conditions such as pelvic inflammatory disease (PID), tubal infertility, and ectopic pregnancies [2,3].

In 2019, 17,718 infections caused by CT were reported in Spain. Around 54.4% of cases were in women. In Spain, these infections were diagnosed in women aged between 25 and 34 years (36.1%), and between 20 and 24 years (27.6%) [4].

Factors associated with CT transmission include having multiple sexual partners, engaging in unprotected sexual activities or having sexual intercourse with symptomatic people, early sexual initiation (under the age of 15 years), or having had previous STI or PID [4]. Moreover, coinfection with another pathogen related to an STI would be an important factor in contracting CT [5]. Finally, taking oral hormonal contraception pills has been linked to rising CT infection rates and it could also lead to more fertility sequelae in women [5].

Most often, CT infection occurs without symptoms, and therefore cannot be diagnosed during its acute period. Its incubation time ranges from five to 10 days after exposure to the pathogen [6]. However, many factors lead CT infection to persist in women’s reproductive tracts such as the CT natural history, specific CT serovar [7], or the ability of CT to evade immune destruction that would cause the infection to persist. Screening and treating for CT infection could prevent the spread of infection and decrease the incidence of infertility. However, some researchers note that conducting screening does not reduce the prevalence of infection [6].

A CT antibody detection is not carried out routinely in Catalonia, Spain for fertility studies because CT is thought to be less prevalent than it is in reality. Our goal was to evaluate whether a previous infection among sexually active women was significantly associated with a diagnosis of infertility and thus be able to recommend policy changes regarding the control of CT infection.

## Materials and methods

This is a prospective study of women who attend an infertility unit in the Lleida Health Region of Spain that serves 400,000 people.

Sample size

We did not calculate the sample size as our goal was to recruit all patients who attended the infertility unit.

Inclusion criteria

All women patients aged over 18 years of age who attend the infertility unit at the gynecology department of a university hospital during 2021. Also included were women who have tried to conceive for one year, or six months in the case of those older than 35 years.

Exclusion criteria

Patients who did not consent to participate in the study.

Variables

To carry out this study, sociodemographic variables, such as age, were collected. Regarding variables related to sexually transmitted diseases, we used the results of routine tests conducted during the fertility study such as antibodies against the hepatitis B virus, antibodies against the hepatitis C virus, human immunodeficiency virus (HIV), syphilis, and human papillomavirus (HPV). After obtaining consent, blood was drawn for immunoglobulin (Ig)G and IgM antibodies for CT.

To evaluate other causes of infertility, we collected levels of follicle-stimulating hormone (FSH), estradiol, thyroid-stimulating hormone (TSH), prolactin, and anti-mullerian hormone. We also included previous gynecological history related to infertility, such as a history of miscarriage, a previous STI, and a history of cervical disease.

Finally, we assessed whether the pregnancy was achieved during 12 months of follow-up and whether the diagnosis of infertility was confirmed.

Statistical analysis

A descriptive statistical analysis was performed using measures of central tendency and dispersion of the quantitative variables consisting of means and standard deviations (SD) for the normal quantitative variables (median and interquartile range (IQR) otherwise) and absolute and relative frequencies for the qualitative variables, comparative to the diagnosis of infertility. It also includes a bivariate analysis of all candidate variables that are potentially explanatory or associated with a diagnosis of infertility or whether the pregnancy was achieved by applying Student's t in the case of normal quantitative candidate variables (Mann-Whitney U otherwise) and the chi-square test for qualitative candidate variables (or Fisher's exact test for expected frequencies below 5). Finally, a logistic regression model was constructed for the diagnosis of female infertility based on the presence of chlamydia antibodies adjusting for all explanatory variables that prove to be important (application of the Borutha algorithm) and contribute significantly (likelihood ratio test (LRT)) to explaining the existence of female fertility problems. The logistic regression model evaluated the calibration (Hosmer-Lemeshow test) and its ability to discriminate (area under the receiver operating characteristic (ROC) curve). Analyses were carried out using the R Project version 4.1.2.

Ethical aspects

Informed consent was obtained from all participants prior to participating in the study. This study was approved by the Clinical Research Ethics Committee of the Hospital Universitario Arnau de Vilanova (HUAV) (approval no.: CEIC-2247). Data were collected for research purposes as per Spanish law 3/2018, on the protection of personal data and the guarantee of digital rights (LOPD-GDD) and regulation 2016/679 of the European Parliament. The study followed the rules of the World Medical Association Declaration of Helsinki.

## Results

Ninety-five patients attended the fertility clinic in 2021. The mean age was 32.2 years. Thirteen patients conceived while 53%) had a final diagnosis of infertility. 

The comparison between the variables we studied and the outcome of the final infertility diagnosis and sample description is shown in Table 1. This table also shows the characteristics of the patients finally diagnosed with infertility (50) vs. those not diagnosed with infertility (45). We did not have not all data from four patients; and for the variable prolactin, we had no data for 47 patients.

**Table 1:** Comparison between the variables and eventual infertility outcome CHAT_IgM: *Chlamydia trachomatis* immunoglobulin M antibodies, CHAT_IgG: *Chlamydia trachomatis *immunoglobulin G antibodies, ASCUS: Atypical squamous cells of undetermined significance, AMH: Antimullerian hormone, HIV: Human immunodeficiency virus, HBsAg: Hepatitis B surface antigen, HVC: Hepatitis C virus, STI: Sexually transmitted infection, HPV: Human papillomavirus, FSH:  Follicle-stimulating hormone, TSH: Thyroid-stimulating hormone, AcHBS: Anti-hepatitis B virus surface antibody + Normal values: FSH 3-9 mUI/ml; TSH 0.2-4.7 mUI/ml; Estradiol 27-161 pg/ml; Prolactin 0-20 ng/ml; AMH 0.7-3.5 ng/ml

	All Variables	No Infertility	Infertility Diagnosed	p-value	N
N (%)		45 (47.4%)	50 (52.6%)		
Mean Age (SD)	32.2 (4.54)	30.7 (4.53)	33.6 (4.12)	0.001	95
CHAT_IgM Levels*	0.26 [0.14;0.48]	0.25 [0.13;0.45]	0.26 [0.18;0.62]	0.369	94
CHAT_IgG Levels*	0.24 [0.14;0.67]	0.22 [0.05;0.40]	0.31 [0.15;1.00]	0.084	94
Syphilis: Negative	92 (100%)	45 (48.9%)	47 (51.1%)	.	92
HIV: Negative	95 (100%)	45 (47.4%)	50 (52.6%)	.	95
HBsAg: Negative	94 (98.9%)	44 (46.8%)	50 (53.2%)	0.474	95
HBsAg: Positive	1 (1.05%)	1 (100%)	0 (0.00%)		
CoreT: Negative	92 (96.8%)	42 (45.7%)	50 (54.3%)	0.103	95
CoreT: Positive	3 (3.16%)	3 (100%)	0 (0.00%)		
AcHBs: Negative	91 (95.8%)	44 (48.4%)	47 (51.6%)	0,619	95
AcHBs: Positive	4 (4.21%)	1 (25.0%)	3 (75.0%)		
HVC Serology: Negative	94 (98.9%)	44 (46.8%)	50 (53.2%)	0,474	95
HVC Serology: Positive	1 (1.05%)	1 (100%)	0 (0.00%)		
Previous miscarriage: No	65 (68.4%)	30 (46.2%)	35 (53.8%)	0.898	95
Previous miscarriage: Yes	30 (31.6%)	15 (50.0%)	15 (50.0%)		
Previous STI: No	68 (71.6%)	33 (48.5%)	35 (51.5%)	0.895	95
Previous STI: Yes	27 (28.4%)	12 (44.4%)	15 (55.6%)		
Previous cervical lesions: No	87 (91.6%)	41 (47.1%)	46 (52.9%)	1	95
Previous cervical lesions: ASCUS	2 (2.11%)	1 (50.0%)	1 (50.0%)		
Previous cervical lesions: Dysplasia	6 (6.32%)	3 (50.0%)	3 (50.0%)		
HPV positive: No	90 (94.7%)	43 (47.8%)	47 (52.2%)	1	95
HPV positive: Yes	5 (5.26%)	2 (40.0%)	3 (60.0%)		
FSH Levels^+ ^mUI/ml	6.90 [5.65;8.40]	6.70 [5.50;7.90]	7.15 [5.73;8.75]	0.163	91
Estradiol Levels^+^ pg/ml	0.14 [0.10;0.21]	0.14 [0.10;0.21]	0.14 [0.10;0.20]	0.99	90
TSH Levels^+^ mUI/ml	2.46 [1.74;3.36]	2.53 [1.88;3.10]	2.33 [1.32;3.60]	0.375	85
Prolactin Levels^+^ ng/ml	20.0 (6.91)	19.4 (7.91)	20.6 (5.88)	0.584	44
AMH* Levels^+^ ng/ml	2.27 [1.31;3.54]	2.71 [1.56;3.31]	2.09 [1.29;4.09]	0.326	80

Among women diagnosed as fertile or infertile, only their age showed statistically significant differences, with higher mean age (33.6 (SD=4.12) years) for women diagnosed with infertility in comparison to those who were not (30.7 (SD=4.53)). The study showed differences according to fertility diagnoses approaching statistical significance (p-value < 0.15) for CT IgG levels (p-value 0.084).

**Figure 1:**
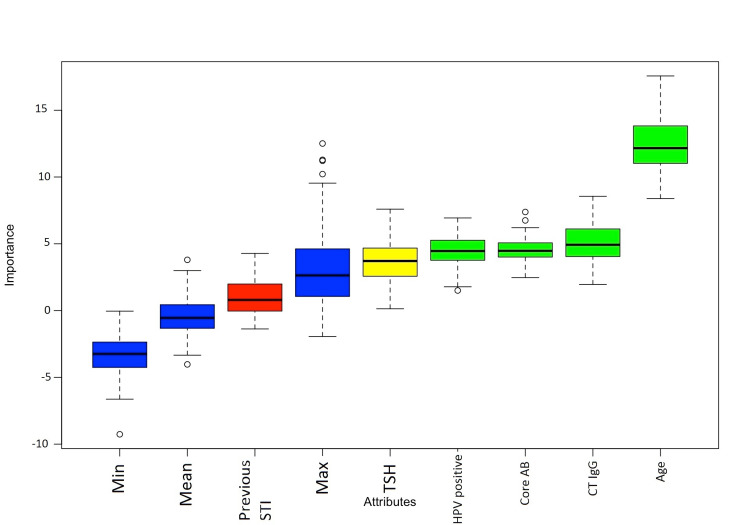
Borutha algorithm to analyze variables related to infertility Min: Minimum, Max: Maximum, STI: Sexually transmitted infection, TSH: Thyroid-stimulating hormone, HPV: Human papillomavirus, AB: Antibody, CT IgG: *Chlamydia trachomatis * immunoglobulin G

Borutha's algorithm detected the following variables as important for infertility: age, IgG antibodies against chlamydia, and a history of HPV (Figure 1). With these variables, a multivariate logistic regression analysis was performed to evaluate these variables' ability to discriminate the diagnosis of infertility. The multivariate logistic regression model keeps just two significant variables: age and IgG antibodies against CT. All the other explanatory variables that prove to be important (application of the Borutha algorithm) didn’t contribute significantly (likelihood ratio test (LRT)) to explaining the existence of female fertility problems.

**Figure 2:**
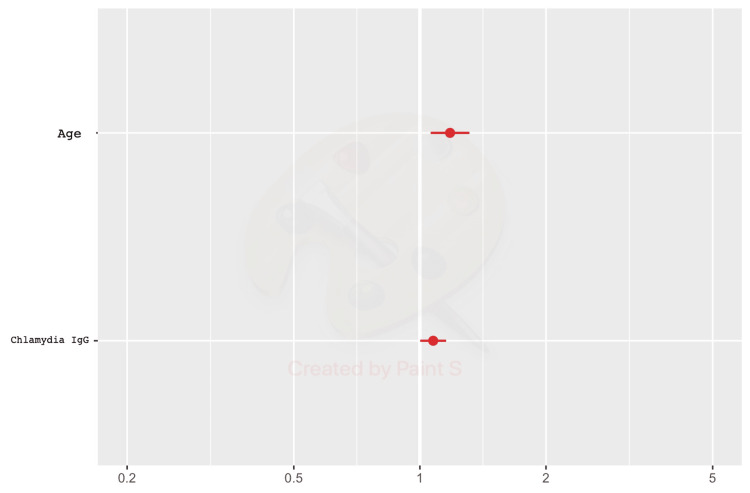
Final multivariable regression model IgG: Immunoglobulin G

Figure 2 shows that the final multivariable regression model included only two significant variables: age (p-value = 0.002) and CT IgG (p-value = 0.045). Thus, the age variable with an odds ratio (OR) = 1.18 and the CT IgG variable with an OR = 1.08. The research concluded that both features were associated with an increased percentage of infertility.

Thirteen female patients (13.7% of the sample) during the follow-up year managed to become pregnant. They accounted for 28.8% of patients who had not been diagnosed as being infertile. Women who conceived displayed significantly lower levels of CT IgG than the other women (with a median of 0.15 vs. 0.27 units). Secondly, the study showed that the women who conceived had significantly lower levels of prolactin (with a mean of 16.6 vs. 20.8 ug/L). 

## Discussion

In this study, we found that having CT antibodies (IgG) was associated with an increased prevalence of infertility. Increasing age was associated with infertility.

Our findings are in line with other studies in this field. In a cohort of Dutch women who had previously tested positive for CT, researchers observed that among this group, the percentage of tubal infertility was higher compared with CT negatives (1.3 per 1000 py (95% confidence interval (CI) 0.8 to 2.1) and 0.2 per 1000 py (95% CI 0.1 to 0.4, respectively). In addition, antibodies for chlamydia were also associated with PID, but not with ectopic pregnancies [6]. Moreover, a study published by the Infectious Diseases Society of America in 2019 with a cohort of 857.324 women, demonstrated that women who tested positive for CT had an increased risk of PID, ectopic pregnancy, and female infertility [7]. However, a study performed by Andersen et al., in Denmark, published in 2010, pointed out that testing young asymptomatic men and women did not reduce long-term reproductive sequelae in either men or women [8]. Nevertheless, Budrys et al. found in 2012 that there were specific chlamydial antigens linked to tubal infertility: heat shock proteins (HSP)60, CT376, CT557, and CT443 [9].

Although there is diverse literature that has studied the impact of CT on fertility, this is the first study to have looked at the association between CT and fertility in Spain. Given the importance and the increase in cases of CT that are being seen in Spain, we have confirmed that in our sample it remains the only STI evaluated that can cause sterility in young women.

Our study has several limitations. Some patients had incomplete data. We were not able to verify the medical history of women recently assigned to our hospital coming from other regions or countries, and if patients did not remember their gynecological history, this information was lost. Finally, we obtained a small sample and the results are from one clinic. 

## Conclusions

Our study has shown an association between a previous CT infection and an increased risk of 12-month adverse fertility. With these results, we believe that it is important to continue working on CT screening projects in asymptomatic young women, to prevent fertility problems and improve women’s reproductive health. 
